# Analysis of the human breast milk microbiome and bacterial extracellular vesicles in healthy mothers

**DOI:** 10.1038/s12276-020-0470-5

**Published:** 2020-08-03

**Authors:** Su Yeong Kim, Dae Yong Yi

**Affiliations:** 1 Department of Pediatrics, Chung-Ang University Hospital, Seoul, 06973 South Korea; 2 College of Medicine, Chung-Ang University, Seoul, 06911 South Korea

**Keywords:** Immunogenetics, Immunization

## Abstract

The microbiota of human breast milk (HBM) contribute to infant gut colonization; however, whether bacterial extracellular vesicles (EVs) are present in HBM or might contribute to this process remains unknown. In this study, we characterized the HBM microbiota of healthy Korean mothers and measured the key bacteria likely affecting infant gut colonization by analyzing both the microbiota and bacterial EVs. A total of 22 HBM samples were collected from lactating mothers. The DNA of bacteria and bacteria-derived EVs was extracted from each sample. In alpha-diversity analyses, bacterial samples showed higher richness and evenness than bacterial EV samples, and beta-diversity analyses showed significant differences between bacteria and bacterial EVs within identical individual samples. Firmicutes accounted for the largest proportion among the phyla, followed by Proteobacteria, Bacteroidetes, and Actinobacteria, in both bacteria and bacterial EV samples. At the genus level, *Streptococcus* (25.1%) and *Staphylococcus* (10.7%) were predominant in bacterial samples, whereas *Bacteroides* (9.1%), *Acinetobacter* (6.9%), and *Lactobacillaceae(f)* (5.5%) were prevalent in bacterial EV samples. Several genera, including *Bifidobacterium*, were significantly positively correlated between the two samples. This study revealed the diverse bacterial communities in the HBM of healthy lactating mothers, and found that gut-associated genera accounted for a high proportion in bacterial EV samples. Our findings suggest the existence of key bacteria with metabolic activity that are independent of the major bacterial populations that inhabit HBM, and the possibility that EVs derived from these bacteria are involved in the vertical transfer of gut microbiota.

## Gut bacteria: Analysing transfer from mother to child

Human breast milk contains a diverse population of bacteria, and tiny membrane-bound packages called extracellular vesicles (EVs) released by bacteria, both of which may contribute to the establishment of beneficial bacterial populations in the infant gut. The microbes that live naturally in our gut are believed to be involved in the development of a healthy immune system, as well as supporting digestion and influencing other aspects of health. Su Yeong Kim and Dae Yong Yi at Chung-Ang University Hospital in Seoul, South Korea, performed a detailed analysis of DNA from bacteria and EVs in breast milk produced by healthy mothers. In addition to categorising the diverse bacterial populations, the study confirms the presence of EVs in human breast milk. These EVs may influence the transmission of bacteria from the mother into an infant’s gut.

## Introduction

More than 100 trillion microbes dwell within the human gut and interact with the host in a symbiotic relationship to aid the development of the host immune system. The early acquisition of the gut microbiome during infancy affects health throughout human life. Several factors, such as the type of delivery, antibiotic therapy, environment of care, and nutritional exposure, are assumed to influence the early colonization of the infant gut^1^. Diet plays a key role in the development of the newborn’s immune system. Human breast milk (HBM) is not only the best source of nutrition for infants, but is also known to contain immune components, such as secretory antibodies, immune cells, antimicrobial proteins (such as lactoferrin and lysozyme), cytokines, and human milk oligosaccharide^2^. Before the 2000s, the presence of bacteria in HBM was thought to indicate contamination or infection, but several researchers have demonstrated that HBM contains commensal bacteria by using culture-dependent techniques^3,4^. The development of the non-culture-based sequencing technique has led to more thorough investigation of the diversity of the HBM microbiota^5^. Currently, it is recognized that HBM contains abundant commensal bacteria that can affect the colonization of the infant gut^6^.

Extracellular vesicles (EVs) are nanometer-sized membrane vesicles that contain various bioactive molecules, such as transmembrane proteins, cytosolic proteins, nucleic acids, and lipids. It is widely accepted that all kinds of bacteria release EVs. Bacteria are classified into Gram-positive (G + ) and Gram-negative (G–) bacteria based on membrane characteristics, and the G + bacteria-derived EVs are usually called bacterial membrane vesicles, while the G-bacteria-derived EVs are called outer membrane vesicles^7^. Bacteria-derived EVs can be detected in body fluids, such as blood, urine, or stool, indicating that they can affect host cells by directly activating host receptors, delivering various bioactive molecules, or integrating EVs into host cells^8–10^. However, there has been no study on the analysis of bacteria-derived EVs in HBM until now.

In this context, the objective of this study was to characterize the HBM microbiota of healthy lactating mothers in Korea, and to measure the abundance of key bacteria that can affect infant gut colonization by analyzing both the microbiota and bacterial EVs using a culture-independent technique.

## Materials and methods

### Subjects and sample collection

This study was conducted according to the guidelines proposed in the Declaration of Helsinki and approved by the Institutional Review Board (IRB) of Chung-Ang University Hospital, Seoul, South Korea (IRB No.: 1810-004-309). Healthy lactating mothers who had delivered a full-term baby were recruited within 1 week–4 months of childbirth for this study (July 2017–June 2018).

Samples of breast milk were collected from 22 mothers in sterile bags by manual or pump expression. Before milk expression, hands were washed, and nipples were cleaned with sterile water. The first ten drops were discarded. The fresh samples were delivered to the laboratory within 24 h and then stored at −80 °C until further processing. If the samples did not reach the laboratory within 24 h, they were excluded. Changes in bacterial diversity based on the lactation stage, especially the difference between the microbiomes of the colostrum and mature HBM, are well known^1^. As we tried to analyze the bacteria that infants actually consume while feeding on stabilized mature milk, we excluded the colostrum samples obtained within 1 week of childbirth. Samples from mothers who developed disease or were administered antibiotics during lactation were also excluded.

### EV isolation and DNA extraction from human milk samples

To separate the EVs from HBM, EVs in milk samples were isolated using differential centrifugation at 10,000×*g* for 10 min at 4 °C. After centrifugation, the pellet contained bacteria, and the supernatant contained EVs. Bacteria and foreign particles were thoroughly eliminated by sterilizing the supernatant through a 0.22-µm filter. To extract the DNA from bacteria and bacterial EVs, bacteria and EVs were boiled for 40 min at 100 °C. To eliminate the remaining floating particles and waste, the sample was centrifuged at 13,000 rpm for 30 min at 4 °C, and the supernatant was collected. DNA was extracted using a DNA isolation kit (DNeasy PowerSoil Kit, QIAGEN, Germany) in accordance with the standard protocol provided along with the kit. The DNA from bacteria and EVs in each sample was quantified using the QIAxpert system (QIAGEN, Germany).

### Bacterial metagenomic analysis using DNA from human milk samples

Bacterial genomic DNA was amplified with the primers 16S_V3_F (5′-TCG GCA GCG TCA GAT GTG TAT AAG AGA CAG CCT ACG GGN GGC WGC AG-3′) and 16S_V4_R (5′-GTC TCG TGG GCT CGG AGA TGT GTA TAA GAG ACA GGA CTA CHV GGG TAT CTA ATC C-3′), which are specific for the V3–V4 hypervariable regions of the 16S rDNA gene. The libraries were prepared using PCR products according to the MiSeq System guide (Illumina, USA) and quantified using QIAxpert (QIAGEN, Germany). Each amplicon was then quantified, set to an equimolar ratio, pooled, and sequenced on a MiSeq instrument (Illumina, USA) according to the manufacturer’s recommendations.

### Analysis of bacterial composition in the microbiota

Paired-end reads that matched the adapter sequences were trimmed by cutadapt version 1.1.6^11^. The resulting FASTQ files containing paired-end reads were merged with CASPER and then quality- filtered using the Phred (Q) score-based criteria described by Bokulich^12,13^. Any reads shorter than 350 bp and longer than 550 bp after merging were also discarded. To identify the chimeric sequences, a reference-based chimera-detection step was performed using VSEARCH against the SILVA gold database^14,15^. Next, the sequence reads were clustered into operational taxonomic units (OTUs) using VSEARCH with a de novo clustering algorithm with a sequence similarity threshold of 97%. The representative sequences of the OTUs were finally classified using the GREENGENES database with UCLUST (*parallel_assign_taxonomy_uclust.py* script on QIIME version 1.9.1) with default parameters^16^.

### Statistical analysis

The richness (rarefaction curves) was analyzed using Chao 1, and alpha-diversity indices (Chao 1, ACE, Shannon index, Simpson index, and Fisher’s index) were calculated. Significance between subgroups was tested using a *t* test for continuous variables. Principal component analysis (PCA) based on Euclidean distance was performed to quantify the relationships between samples. In addition, networks were constructed using the Pearson correlation coefficient. All statistical analyses were performed using R version 3.3.2.

## Results

### Characteristics of the lactating mothers

A total of 22 lactating mothers were enrolled in this study; the mean age was 33.1 ± 3.4 years. Of the 22 lactating mothers, 16 (72.7%) were primiparous, and 6 (27.3%) were multiparous. Eight (36.4%) mothers had vaginal deliveries and 14 (63.6%) mothers had delivered by cesarian section (CS). Samples were collected 11.1 ± 2.4 days after delivery on average.

### Diversity

**Fig. 1:**
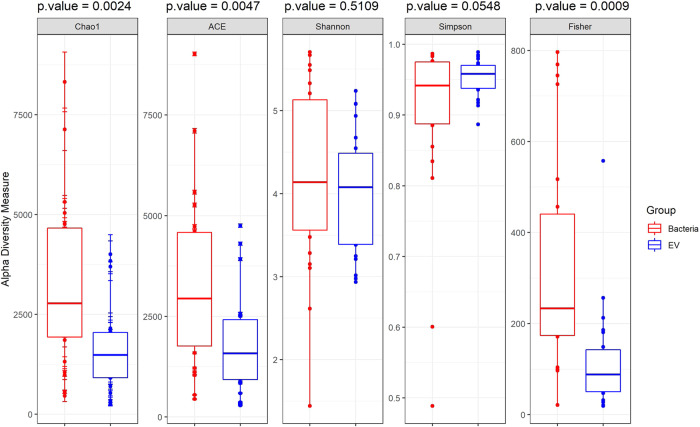
Alpha diversity analysis in bacterial samples and bacterial extracellular vesicle (EV) samples. Alpha diversity analysis showed that microbial richness and evenness were significantly higher in bacterial samples than in bacterial EV samples when analyzed using Chao 1 (*p* = 0.002), ACE (*p* = 0.005), and Fisher’s exact test (*p* < 0.001). Bacteria and bacterial EV samples did not show any significant difference in Shannon diversity index (*p* = 0.51) or Simpson’s index (*p* = 0.05).

When Chao 1 and ACE were determined to analyze the alpha diversity and richness, bacteria showed significantly higher alpha diversity than bacterial EVs (*p* < 0.001 and *p* = 0.005, respectively). Bacteria showed higher Fisher’s index values than the EVs, indicating higher evenness in the bacterial samples (*p* < 0.001). However, EVs and bacteria did not show any significant difference in the Shannon diversity index (*p* = 0.51), which signifies richness, and the Simpson index (*p* = 0.05), which signifies evenness (Fig. 1). The number of reads for bacteria, 14966.6 (SD 10189.1), was higher than that for EVs, 9267.8 (SD 6507.0). Bacteria also showed significantly higher levels of operational taxonomic units (OTUs), with 1202.3 (SD 789.1) reads compared with 501.0 (SD 446.8) reads in EVs.

**Fig. 2:**
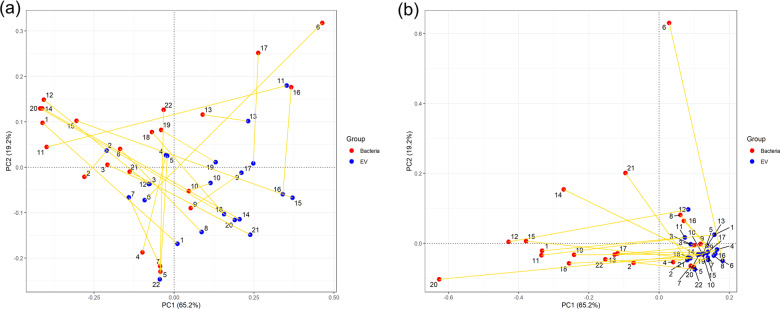
Beta-diversity analysis using PCA based on the Euclidean distance. (**a**) at the phylum level and (**b**) at the genus level.

The beta diversity of all samples was analyzed using PCA based on the Euclidean distance (Fig. 2). The Euclidean distance value and cluster in the breast milk samples did not differ significantly between different individual samples at the EV level. However, the Euclidean distance values showed a significant difference in different individual samples at the bacterial level. Furthermore, the Euclidean distance of bacteria and EVs from an identical sample showed a significant difference.

### Bacterial composition

**Fig. 3:**
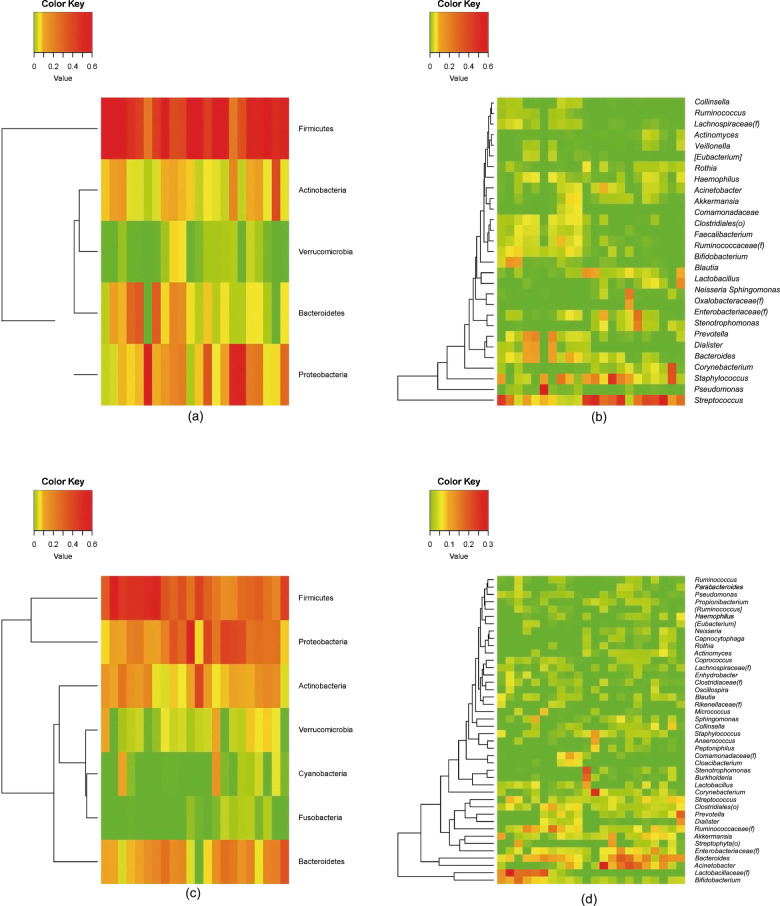
The composition of the human breast milk microbiota in this study. (**a**) Bacteria at the phylum level, (**b**) bacteria at the genus level, (**c**) bacterial extracellular vesicles (EVs) at the phylum level, and (**d**) bacterial EVs at the genus level.

We detected 23 phyla by analyzing the microbiota originating from breast milk. Among the microbes, Firmicutes accounted for the largest proportion, with 56.4% (SD 19.4) among the phyla, followed by Proteobacteria (19.6% (SD 19.1)), Bacteroidetes (9.8% (SD 10.5)), and Actinobacteria (9.0% (SD 7.9)). Verrucomicrobia showed an average proportion below 5%, while other phyla accounted for <1% of the bacterial composition of breast milk (Fig. 3a). Three-hundred and ninety-two genera were detected in total at the genus level, with *Streptococcus* accounting for the highest proportion at 25.1% (SD 20.9), followed by *Staphylococcus* at 10.7% (SD 12.3). *Bacteroides*, *Acinetobacter*, Enterobacteriaceae(f), Ruminococcaceae(f)*, Bifidobacterium, Prevotella*, Clostridiales(o)*, Corynebacterium, Akkermansia, Lactobacillus, Pseudomonas, Dialister, Stenotrophomonas, Blautia, Sphingomonas, Haemophilus, Neisseria*, Lachnospiraceae(f)*, Rothia*, and *Faecalibacterium* showed average proportions below 5%, while other genera accounted for <1% of the bacterial composition on average (Fig. 3b).

Meanwhile, bacterial EV analysis revealed a total of 22 phyla. Firmicutes accounted for the largest proportion (35.8% (SD 12.9)), while Proteobacteria (24.5% (SD 13.3)), Bacteroidetes (15.5% (SD 8.8)), and Actinobacteria (13.1% (SD 8.3)) accounted for the remaining majority of the bacterial EV composition. Verrucomicrobia and Cyanobacteria accounted for less than 5% of the bacterial EV microbiome on average, and other phyla accounted for less than 1% on average (Fig. 3c). A total of 291 genera were detected at the genus level in bacterial EV samples. *Bacteroides, Acinetobacter*, and *Lactobacillaceae(f)* accounted for high proportions of EV samples (9.1% (SD 5.4), 6.9% (SD 8.2), and 5.5% (SD 9.1), respectively). Meanwhile, *Streptococcus, Staphylococcus*, Enterobacteriaceae(f), Ruminococcaceae(f), *Bifidobacterium, Prevotella*, Clostridiales(o)*, Corynebacterium, Akkermansia, Lactobacillus, Dialister, Stenotrophomonas, Blautia, Sphingomonas*, Streptophyta(o), Comamonadaceae(f), *Collinsella, Actinomyces*, Clostridiaceae(f), and *Burkholderia* accounted for less than 5% of the bacterial EV composition on average. The rest of the detected genera accounted for <1% of the bacterial EV composition on average (Fig. 3d).

### Correlation between bacteria and bacterial EVs

**Fig. 4:**
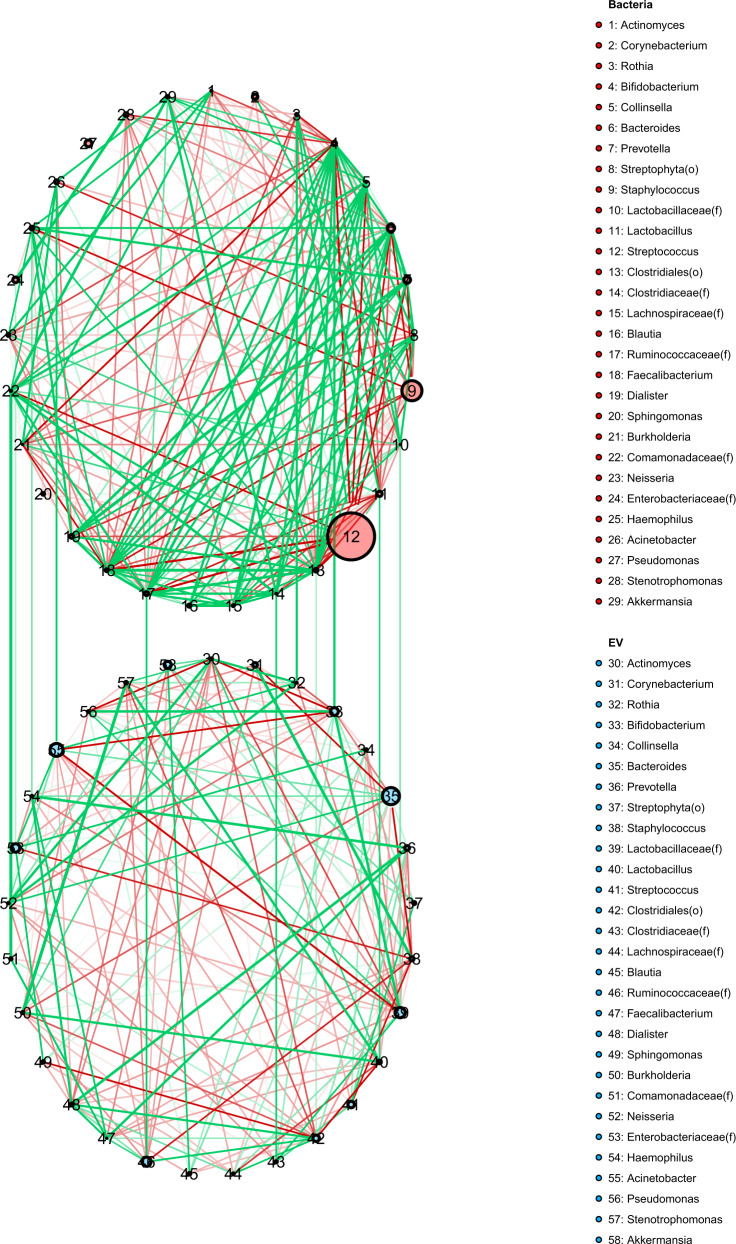
Correlation analysis between bacteria and bacterial extracellular vesicle (EV) samples. Correlation analysis between bacteria and bacterial EV samples showed a significant positive correlation for Acinetobacter, Ruminococcaceae (**f**), Bifidobacterium, Comamonadaceae (**f**), Rothia, and Clostridiaceae (**f)** (*p* < 0.05) (see also Supplementary Table S1).

We analyzed the correlation between bacteria isolated from HBM (bacteria) and bacterial EVs at the phylum level, and determined that there were no taxa with a significant correlation between the bacteria and bacterial EV samples. However, at the genus level, we did observe significant differences in the proportions of several genera. We analyzed these correlations to determine any trends, and found that *Acinetobacter*, Ruminococcaceae(f), *Bifidobacterium*, Comamonadaceae(f), *Rothia*, and Clostridiaceae(f) showed a significant positive correlation between bacteria derived from HBM and bacterial EV samples (*p* < 0.05) (Fig. 4; Supplementary Table S1).

Comamonadaceae(f) showed a particularly high correlation between the two sample groups (*r* = 0.95). Although they account for less than 1% of the total proportion, *Comamonas* and *Diaphorobacter* also showed a significantly high correlation between bacteria and bacterial EV samples (*r* = 0.94). Among the bacteria accounting for <1% of the total proportion, 24 genera showed a significant positive correlation. Meanwhile, *Bacteroides, Prevotella*, Lachnospiraceae(f), *Collinsella*, and *Burkholderia* showed a negative correlation trend, although this trend was not significant. Although some genera accounted for less than 1% of the average proportion with a negative correlation, this result was not significant.

### Differences between subgroups

**Fig. 5:**
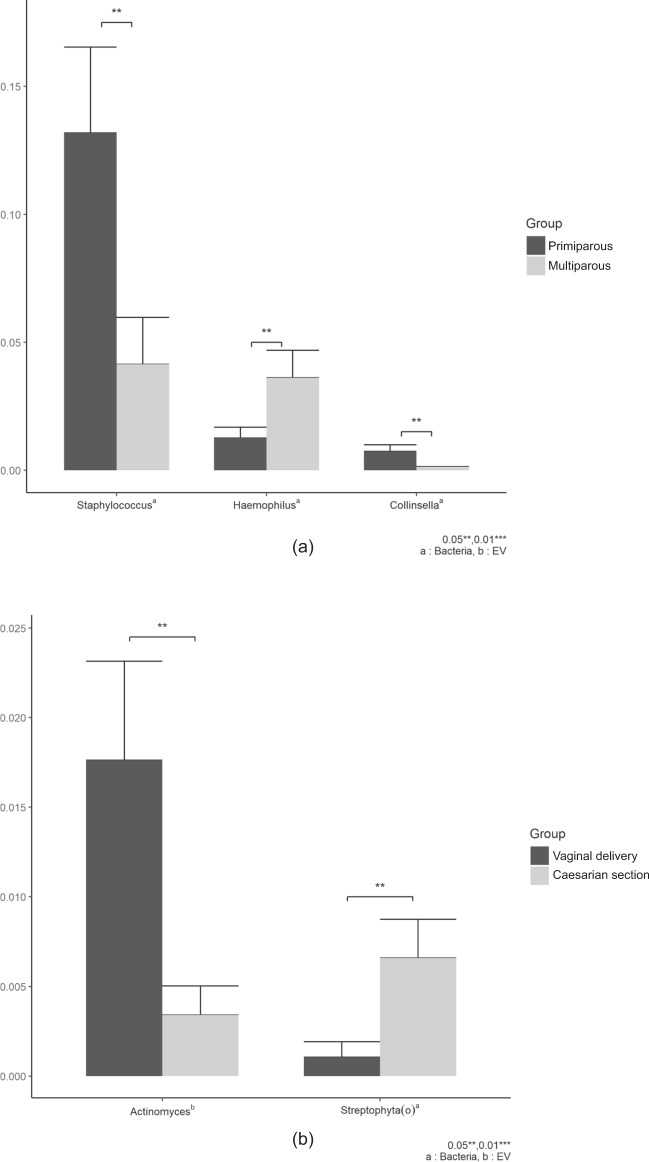
Subgroup analysis of the bacterial composition of human breast milk. Analysis of bacterial composition based on (**a**) parity and (**b**) mode of delivery.

We also analyzed the bacterial composition of HBM at the genus level based on parity. We determined 0.31- and 0.18-fold changes between bacteria and bacterial EV samples with respect to *Staphylococcus* and *Collinsella*, respectively, which are significantly lower than the values obtained when the breast milk was obtained from multiparous mothers. Meanwhile, the abundance of *Haemophilus* was shown to be 2.86-fold higher in the HBM of multiparous mothers than in that of primiparous mothers (*p* < 0.05) (Fig. 5a). No significant difference in the microbiome based on parity was observed in bacterial EV samples. When we compared the microbial composition of HBM of mothers who delivered by CS with that of mothers who had vaginal delivery, the abundance of Streptophyta(o) was significantly higher (6.24-fold) in bacterial samples from mothers who delivered by CS (Fig. 5b). The abundance of Actinomycetales was significantly lower (0.19-fold) in bacterial EV samples from mothers who delivered by CS than in those from mothers who had vaginal delivery (Fig. 5b). A comparative analysis was conducted comparing calories; however, none of the microbiomes showed any significant difference.

## Discussion

To the best of our knowledge, this is the first study to analyze both the microbiota and bacteria-derived EVs in HBM using next-generation sequencing (NGS) worldwide. In the present study, we found that HBM of healthy lactating mothers contained diverse and complex bacterial communities, with Firmicutes as the predominant phylum. An analysis at the genus level showed significant differences in proportions between the microbiota and bacteria-derived EV samples, and several genera showed a significant positive correlation between the microbiota and bacterial EVs.

An initial study that analyzed the HBM using NGS suggested that there was a common core microbiome consisting of nine bacterial genera^17^. Several subsequent studies also identified the bacteria that make up the core HBM microbiome. However, the results differed from study to study^6,18,19^. The core HBM microbiome has been the subject of controversy, as it is thought to play a major role in the colonization of the infant gut. A systematic review analyzed 242 studies and found that most of the bacterial species isolated from breast tissue and milk belonged to four bacterial phyla, namely, Firmicutes, Proteobacteria, Actinobacteria, and Bacteroidetes^20^. A recent study, which reviewed 44 studies that investigated 3105 breast milk samples from 2655 women, showed that the most frequently found genera were *Staphylococcus, Streptococcus, Lactobacillus, Pseudomonas, Bifidobacterium, Corynebacterium, Enterococcus, Acinetobacter, Rothia, Cutibacterium, Veillonella*, and *Bacteroides*^21^. Another systematic review of 12 studies that used only culture-independent methods for healthy mothers reported that *Staphylococcus* and *Streptococcus* were the predominant genera, and that these genera may be universally present in HBM^5^. In our study, Firmicutes accounted for the largest proportion among the phyla, followed by Proteobacteria, Bacteroidetes, and Actinobacteria, in both bacteria and bacterial EV samples. At the genus level, *Streptococcus* (25.1%) and *Staphylococcus* (10.7%) were predominant in bacterial samples. The results of the present study are in accordance with those of previous studies. However, analysis at the genus level showed significant differences in proportions between bacteria and bacterial EVs.

As no research has been done to analyze bacteria-derived EVs in HBM, EVs released from the HBM microbiota are bound to be a part of the findings. A study has reported that intraperitoneally injected bacterial EVs spread throughout the mouse body, accumulating in the liver, lung, spleen, and kidney within 3 h of administration^22^. Several studies have also reported that bacteria-derived EVs can be detected in human blood serum, urine, or stool, and have claimed that bacteria-derived EVs are related to the immune response or development of disease^23–27^. For example, studies have shown that EVs derived from *Staphylococcus aureus* contribute to atopic dermatitis-like skin inflammation^28,29^. In the present study, we were able to detect bacterial EVs in HBM, and unlike the dominant genera in the bacterial sample, *Bacteroides* (9.1%), *Acinetobacter* (6.9%), and Lactobacillaceae(f) (5.5%) accounted for a high proportion. When we analyzed the alpha diversity, the present study showed that the number of reads and OTUs in bacterial samples were higher than those in bacterial EVs. Bacterial samples also showed higher richness and evenness than bacterial EV samples. Beta-diversity analyses showed significant differences between bacteria and bacterial EVs within identical individual samples. The results of the present study indicate that while abundant bacterial communities are distributed evenly in HBM, the bacteria that release EVs do not necessarily match the bacteria present in HBM in high proportion and are not distributed evenly.

The origins of bacteria in HBM are still unclear. It has been suggested that commensal bacteria from maternal skin (such as *Staphylococcus*, *Corynebacterium,* and *Cutibacterium*) or the infant’s mouth (such as *Streptococcus*) can enter the mammary duct during breastfeeding^30^. Another hypothesis is that the maternal gut bacteria enter the mammary glands through the entero-mammary pathway, which would require the cells to penetrate the intestinal epithelium and reach the mammary glands through the bloodstream^31–33^. The observation that anaerobic bacteria such as *Bacteroides*, *Bifidobacterium*, *Parabacteroides*, and *Clostridium* that are not found on the skin are detected and shared between HBM and infant feces supports this hypothesis^3,6^. Other observations showing that there are bacteria in the maternal gut, infant’s gut, and HMB that share the same strains also support the vertical transmission of maternal commensal bacteria to the infant’s gut through HBM^31,34,35^, but no exact mechanism has been revealed. It is thought that not only pathogens but also commensal bacteria can secrete EVs. One study revealed that commensal bacteria can produce EVs containing immunomodulatory molecules and affect host immunity and health^36^, while others suggested that EVs derived from commensal bacteria can be involved in gut colonization^37^. In our study, gut-associated genera such as *Bacteroides* and Lactobacillaceae(f) accounted for a high proportion in the bacterial EV samples, unlike the predominant bacteria in the bacterial samples. Our findings suggest that EVs derived from gut bacteria may contribute to the vertical transfer of the commensal microbiota from mothers to infants via HBM.

Evaluation of the correlation between HBM-derived bacteria and bacterial EV samples revealed that several genera were significantly positively correlated. As a strong correlation can be interpreted as being indicative of high metabolic activity of the bacteria, the effect of these bacteria on gut colonization is worth considering. In particular, we highlighted *Bifidobacterium*, as the gut microbiota of infant feces usually exhibits a high abundance of this bacterium, with exclusively breastfed infants in particular exhibiting a relatively high abundance of *Bifidobacterium* in feces^38,39^. Several lines of evidence suggest that the relative level of *Bifidobacterium* abundance is associated with the development of disease or immunity. For example, a systematic review compared 101 studies on healthy controls with 147 studies on infected patients, all of which included molecular analysis of HBM. Some species were found only in infected patients, whereas others were never detected in infected patients and were present only in healthy controls; specifically, *Bifidobacterium* and *Lactobacillus* were associated with the absence of infection^20^. In this study, *Lactobacillus* was among the bacteria present at high abundance in bacterial EV samples, and *Bifidobacterium* was identified as being highly correlated between bacteria and bacterial EVs. Our findings suggest the presence of key bacteria with significant metabolic activity that are independent of the major bacterial populations that inhabit HBM, and the possibility of measuring the association of these bacteria with EVs. Together, these findings warrant additional research to explore the effects and functions of bacterial EVs on infant gut colonization and immune development.

Various factors, such as mode of delivery, maternal weight, antibiotics, lactation stage, gestational age, geographical location, and maternal health, have been found to contribute to the diversity of the HBM microbiota^1^. In the present study, subgroup analysis revealed that the abundance of *Staphylococcus* in the bacterial samples and *Collinsella* in the bacterial EV samples was significantly lower, and that of *Haemophilus* in the bacterial samples was significantly higher, when HBM was obtained from multiparous mothers compared with primiparous mothers. In contrast, a large study did not identify an effect of parity on the composition of the HBM microbiota^40^. Our study also showed that the abundance of Streptophyta(o) was significantly higher in the bacterial samples, and that of *Actinomycetales* was significantly lower in the bacterial EV samples in the CS than in the vaginal delivery group. It has been suggested that labor is associated with the composition of the HBM microbiota via increased intestinal permeability, and by facilitating a greater degree of entero-mammary transfer of maternal gut bacteria^41^. Women who delivered by CS were reported to have a higher relative abundance of Proteobacteria and *Carnobacteriaceae*, but a lower relative abundance of *Bifidobacterium*, *Leuconostocaceae*, and *Lactobacillus*^1^. Alternatively, some studies have found no association between the composition of the HBM microbiota and delivery mode^40,42^. We therefore postulate that the diversity of bacterial communities is affected by a number of variables, such as host immunity, environmental factors, and microbial factors, resulting in quantitative and qualitative differences.

Our study has some limitations. First, the sample size was relatively small. Second, we did not analyze communities from maternal and infant mouths, skin, and stool simultaneously, so the origins of the HBM bacteria are unclear. Third, we also did not demonstrate the process by which the HBM microbiota and EVs are involved in the colonization of the infant gut. Considering that bacteria reaching the infant’s gut can affect immunity, it is more important to know which bacteria have active biological functions than to simply describe the composition of the HBM microbiota. It is necessary to determine whether EV analysis can predict the key bacteria that play a major role in the colonization of the infant gut, and to identify the factors that influence the release of bacterial EVs in HBM. Studies are also needed to explain how bacterial EVs in HBM affect the composition of the infant gut microbiota and change infant health outcomes. This will be the next task in our future study. Despite these limitations, the present study was the first to analyze bacterial EVs in HBM, suggesting the possibility that bacterial EVs in HBM contribute to the colonization of the infant gut, and will be the basis for further study.

In conclusion, the present study revealed the diverse bacterial communities in HBM from healthy lactating mothers along with those of bacterial EVs and the relationship between these factors. Our findings suggest the existence of key bacteria with metabolic activity in HBM, and the possibility that EVs derived from these bacteria are involved in the vertical transfer of the gut microbiota from mothers to their progeny. Future studies will be needed to confirm the role of bacterial EVs in HBM colonization of the infant gut, and to ultimately promote colonization with beneficial bacteria in HBM.

## Supplementary information

certificate of English editing
