# Supplementary material for: Analysis of the human breast milk microbiome and bacterial extracellular vesicles in healthy mothers
Source: Exp Mol Med. 2020 Aug 3;52(8):1288–97. doi: 10.1038/s12276-020-0470-5 (PMC8080581; doi:10.1038/s12276-020-0470-5)
Supplement: Supplementary file 1 — certificate of English editing [file 12276_2020_470_MOESM1_ESM.pdf]

TABLE S1 Correlation between the taxa present in bacteria and bacterial extracellular vesicle samples.

	**Bacteria**		**Extracellular vesicles**		**Pearson correlation**	
**Taxon**	**mean**	**SD**	**mean**	**SD**	**Correlation**	***p*-value**
Streptococcus	0.2509	0.2092	0.0348	0.0280	0.0231	0.919
Bacteroides	0.0392	0.0394	0.0911	0.0542	−0.1276	0.575
Staphylococcus	0.1071	0.1227	0.0220	0.0267	0.1308	0.562
Acinetobacter	0.0202	0.0232	0.0691	0.0815	0.5414	0.009
Enterobacteriaceae(f)	0.0299	0.0584	0.0450	0.0360	0.2586	0.245
Ruminococcaceae(f)	0.0203	0.0243	0.0489	0.0447	0.4867	0.022
Bifidobacterium	0.0224	0.0242	0.0418	0.0464	0.6676	<0.001
Prevotella	0.0356	0.0579	0.0250	0.0421	−0.1578	0.483
Lactobacillaceae(f)	0.0015	0.0030	0.0545	0.0905	0.3049	0.168
Clostridiales(o)	0.0194	0.0225	0.0346	0.0259	0.2678	0.228
Corynebacterium	0.0289	0.0786	0.0244	0.0534	−0.0558	0.805
Akkermansia	0.0132	0.0203	0.0379	0.0323	0.1152	0.609
Lactobacillus	0.0275	0.0395	0.0191	0.0270	0.3725	0.088
Pseudomonas	0.0362	0.1521	0.0093	0.0090	−0.0262	0.908
Dialister	0.0205	0.0398	0.0155	0.0319	−0.0499	0.825
Stenotrophomonas	0.0202	0.0469	0.0138	0.0453	−0.0481	0.831
Blautia	0.0165	0.0385	0.0135	0.0142	−0.0324	0.886
Sphingomonas	0.0144	0.0498	0.0134	0.0228	−0.0532	0.814
Haemophilus	0.0191	0.0219	0.0077	0.0134	0.3138	0.155
Neisseria	0.0155	0.0388	0.0070	0.0106	−0.0473	0.834
Streptophyta(o)	0.0038	0.0060	0.0157	0.0336	0.0359	0.874
Lachnospiraceae(f)	0.0103	0.0130	0.0087	0.0090	−0.1354	0.548
Comamonadaceae(f)	0.0068	0.0175	0.0119	0.0282	0.9507	<0.001
Collinsella	0.0057	0.0091	0.0116	0.0200	−0.1349	0.55
Rothia	0.0120	0.0156	0.0047	0.0094	0.6018	0.003
Faecalibacterium	0.0149	0.0191	0.0014	0.0024	0.1400	0.534
Actinomyces	0.0051	0.0086	0.0105	0.0150	0.1149	0.611
Clostridiaceae(f)	0.0038	0.0059	0.0108	0.0133	0.4420	0.039
Burkholderia	0.0021	0.0040	0.0114	0.0383	−0.1177	0.602
